# Mitigating antimicrobial resistance in the animal industry: reduction strategies and alternatives to antibiotics

**DOI:** 10.3389/fvets.2026.1912728

**Published:** 2026-08-14

**Authors:** Hanan Al-Khalaifah, Tahani Al-Surrayai

**Affiliations:** Environment and Life Sciences Research Center, Kuwait Institute for Scientific Research, Kuwait, Kuwait

**Keywords:** animal industry, antibiotic alternatives, antibiotic reduction strategies, antibiotic resistance, antimicrobial resistance, food-producing animals, livestock production, one health

## Abstract

Antimicrobial resistance (AMR) has become a worldwide issue of human and animal health, food security, and environmental sustainability due to its seriousness and the challenge of resolving it. Widespread utilization of antibiotics in the animal industry for metaphylactic, therapeutic, production, and prophylactic purposes has significantly contributed to the spread and emergence of antibiotic-resistant microorganisms. Food- producing animals are also deemed as good reservoirs of “antimicrobial resistance genes (ARGs) and antimicrobial- resistant bacteria (ARB)” that may contaminate humans through the food chain, environmental pathway, and direct contact. Moreover, the discharge of antibiotic residues into the soil and water systems enhances selective pressure among microbial communities, making AMR a nature of One Health. This review summarizes the major strategies to reduce antibiotics use in the animal industry, which include: better husbandry and biosecurity measures, vaccination, wise use of antimicrobials, manure and environmental management. Special attention is given to non-antibiotic options like probiotics, prebiotics, phytogenic compounds, organic acids, immunomodulators, and other feed-based or biological interventions that are emphasized to maintain animal health and productivity while reducing antibiotic use. Through a combination of these strategies in a One Health paradigm, this review outlines feasible and viable solutions to help curb the problem of AMR in the animal sector. Findings highlight the importance of concerted action by producers, veterinarians, policymakers, and researchers to decrease the reliance on antibiotics and protect human health and animal production systems.

## 1 Introduction

Antimicrobial resistance (AMR) has become one of the most serious global threats to human and animal health, food security, and environmental sustainability. AMR encompasses resistance to antimicrobial agents, including in bacteria, viruses, parasites, and fungi. Antibiotics constitute the largest class of antimicrobials used in food-producing animals and are the principal drivers of resistance development in livestock production. Therefore, the widespread emergence of resistant microorganisms in these systems exemplifies the One Health concept.

Many of the same antibiotic classes, including tetracyclines, penicillins, macrolides, aminoglycosides, sulfonamides, and fluoroquinolones, are used in both human and veterinary medicine. Consequently, antibiotic resistance (AR) arising in food-producing animals may compromise the effectiveness of medically important antibiotics used in human healthcare (1–3).

Since their discovery, antibiotics have been essential for the treatment and prevention of livestock diseases, thereby sustaining global food production (4, 5). In livestock and poultry production systems, antibiotics are used for therapeutic, metaphylactic, prophylactic, and growth-promoting purposes. In the developed world, approximately 50%−80% of antibiotics are utilized in animal agriculture, particularly in swine and poultry production (6, 7). Bacterial pathogens exhibit high levels of resistance to commonly used antibiotic classes, including tetracyclines, sulfonamides, and penicillins (8). However, this extensive use raises public health concerns, as residues may accumulate in milk, meat, and eggs, leading to prolonged human exposure.

The consequences of antibiotic use extend beyond food safety into human health and environmental sustainability. Numerous ARB have been isolated from foods of animal origin, including methicillin-resistant *Staphylococcus aureus* (MRSA), multidrug-resistant (MDR) *E coli, Salmonella* spp., *Campylobacter* spp., *Enterococcus* spp., and extended-spectrum β-lactamase-producing *Enterobacteriaceae* (9). These resistant pathogens may be transmitted to humans through the food chain, direct animal contact, or environmental exposure. In the US alone, more than two million people acquire antimicrobial-resistant bacterial infections annually, resulting in approximately 23,000 deaths (10). Globally, AMR is projected to cause up to 10 million deaths annually by 2050 and impose an economic burden approaching US$100 trillion if effective mitigation strategies are not implemented (11). Recognizing the urgency of this crisis, the World Health Organization (WHO) published its priority list of antibiotic-resistant bacterial pathogens in 2017 to guide research and development of new antimicrobial therapies (12). However, AMR is a multifactorial phenomenon and is not driven solely by antimicrobial use (AMU). Other important contributors include horizontal gene transfer among microorganisms, inadequate infection prevention and biosecurity, environmental dissemination of antimicrobial residues, resistant bacteria and resistance genes, co-selection by heavy metals and biocides, intensive livestock production systems, and insufficient surveillance and regulatory oversight. These interconnected factors facilitate the emergence and spread of AMR and highlight the importance of adopting a comprehensive One Health approach to mitigation (1).

Beyond direct health impacts, the extensive application of antimicrobials has caused environmental pollution through excreta and agricultural or aquaculture wastes (13, 14). Even though most antimicrobials are characterized by short half-lives, their continuous misuse has resulted in the accumulation of residues and degradation products in groundwater, wastewater, surface waters, and even vegetables (6, 15). These residues and their degradation products exhibit ecotoxicity at sub-lethal concentrations, disrupting microbial community structures, affecting aquatic and soil ecosystems, and posing risks to non-target organisms (16–20). Consequently, mitigation strategies must extend beyond prudent antibiotic use to include effective environmental management within the livestock–environment interface.

Addressing this challenge requires a multifaceted approach. While the cautious use of veterinary antibiotics remains essential, it must be augmented with alternative strategies, including probiotics, prebiotics, organic acids, phytochemicals, feed enzymes, and biological interventions (21). These alternatives seek to ensure productivity by improving gut health, immune function, and disease resistance without exerting selective pressure for resistance. The need to reduce dependence on antibiotics is gaining growing global interest, while acknowledging the vital role antibiotics continue to play in animal health.

Although several reviews have been conducted and published on AMR, stewardship and alternative treatment options such as probiotics, phytogenic feed additives or bacteriophages, each of them concentrated mainly on one particular intervention, species or aspect of AMR. The current review presents a comprehensive overview of the One Health perspective on the control and management of AMR. It discusses the available knowledge and experience on appropriate use of antimicrobials, measures related to animal husbandry and biosecurity, vaccination, manure and farm management, regulation and surveillance as well as antimicrobial substitutes in poultry, pigs, cattle, small ruminants, and fish. Unlike previous reviews, it compares the evidence on the effectiveness of currently available interventions and highlights the mechanisms of action, limitations, and applicability of recently proposed treatment options and platforms, including bacteriophages, antimicrobial peptides, quorum-sensing inhibitors, phage-encoded CRISPR-Cas interference, nanotechnology, and artificial intelligence. This review also identifies knowledge gaps and research priorities related to AMR management in food-producing animals. By summarizing the information on antimicrobial use and resistance in a wide range of production animals and discussing the mechanisms of action, effectiveness, and limitations of various interventions, it will serve as a useful guide for scientists, veterinarians, livestock producers and policymakers.

## 2 Methodology

This review was conducted using a structured narrative synthesis approach to examine current knowledge on AMR in animal production systems, with particular emphasis on interventions aimed at reducing AMU and promoting non-antibiotic alternatives. A comprehensive literature search was performed using major scientific databases, including Scopus, PubMed, Web of Science, and Google Scholar. The search was further supplemented with guidelines, policy documents, and technical reports published by international organizations, including the Food and Agriculture Organization (FAO), the World Organization for Animal Health (WOAH), and the WHO.

Keywords used in the search included combinations of AMR, AMU, antibiotic reduction strategies, prudent antimicrobial use, biosecurity, vaccination, animal husbandry, livestock production, poultry production, probiotics, prebiotics, phytogenics, alternatives to antibiotics, and sustainable livestock systems. The search focused primarily on peer-reviewed journal articles, review papers, and authoritative reports published between 2010 and 2026, with particular emphasis on studies published during the last 5 years to capture recent developments in antimicrobial stewardship, resistance trends, and mitigation strategies. No country- or region-specific restrictions were applied during the literature search, enabling inclusion of studies from diverse geographic regions to provide a comprehensive global perspective on AMR and mitigation strategies in food-producing animals.

The initial search yielded a broad collection of publications, which were screened based on title and abstract relevance. After removing duplicate and unrelated studies, full-text articles were assessed for their relevance to AMR management in food-producing animals. Studies were prioritized based on scientific rigor, methodological quality, practical applicability, citation impact, and contribution to understanding AMR reduction strategies. Particular emphasis was placed on research involving poultry and intensive livestock production systems, including studies addressing prudent antimicrobial use, vaccination programs, biosecurity measures, husbandry improvements, environmental management, and nutritional or biological alternatives to antibiotics.

Articles focusing primarily on non-food-producing animals were excluded. However, studies involving companion animals or other non-food-producing species were considered when they provided relevant evidence on AMR mechanisms, antimicrobial stewardship, or One Health concepts that informed the broader understanding of AMR mitigation in food-producing animal systems. To ensure balanced interpretation, conflicting findings across studies were critically evaluated by considering differences in study design, animal species, production systems, geographic regions, intervention types, and management practices.

As this review followed a narrative rather than a quantitative meta-analytical approach, findings were synthesized thematically. The selected literature was organized into key thematic areas, including patterns and drivers of AMU, mechanisms and impacts of AMR, antimicrobial stewardship strategies, biosecurity and management interventions, vaccination and disease prevention, nutritional and biological alternatives to antibiotics, environmental considerations, and future directions for sustainable livestock production.

## 3 Antibiotic use in animal agriculture and its role in the development and spread of AMR

### 3.1 Patterns and drivers of use

Antibiotics are essential in veterinary medicine for treating bacterial infectious diseases in livestock, companion animals, and aquaculture. Their judicious use is critical to preserve efficacy in both veterinary and human medicine (22). Penicillin treats bovine mastitis, pneumonia in young cattle, and erysipelas in swine. Dihydrostreptomycin, a form of streptomycin, has been employed to treat bovine mastitis, pneumonia, leptospirosis, and intrauterine infections. Oxytetracycline and chlortetracycline have been extensively utilized for the prevention of cattle pneumonia, diarrhea in young calves and piglets, and *Pasteurella multocida* infections in birds. Tetracyclines, macrolides, and penicillins have also been used intermittently as growth promoters in pigs, poultry, and calves (23).

Eagar et al. (24) reported that the antimicrobial classes most frequently used in animal agriculture were macrolides and pleuromutilins, followed by tetracyclines, sulfonamides, and penicillins. Approximately 68.5% are administered through feed, 17.5% by parenteral injection, and about 12% via drinking water. These administration routes support therapeutic treatment, disease prevention, and growth promotion, contributing to the economic sustainability of livestock production (22).

Additionally, off-label use of antimicrobials results in increased or decreased absorption, distribution, or excretion of the antimicrobial active ingredient. This is also linked to the observation that as animals grow larger, the proportion of body surface area to body weight diminishes, leading to slower physiological functions than those found in smaller animals. These factors collectively alter drug absorption, distribution, metabolism, and elimination in various species (25). Using antibiotics that are ineffective against the labeled bacteria, or when the dosage on the label is insufficient (due to AMR), results in unnecessary exposure to antibiotics and contributes to AMR (26). In Southeast Asian and African veterinary contexts, empirical use without confirmatory diagnosis—particularly penicillins, lincosamides, quinolones, and sulfonamide-trimethoprim combinations—accounts for a substantial proportion of antibiotic administration (25).

Collectively, extensive therapeutic, prophylactic, metaphylactic, and growth-promoting antibiotic use increases selective pressure on bacterial populations, accelerating the emergence and dissemination of antimicrobial-resistant organisms within animal production systems. Once resistant bacteria emerge within animal production systems, they can spread through multiple transmission pathways, posing significant risks to public health and environmental sustainability.

### 3.2 Transmission of resistant bacteria and public health impact

**Figure 1:**
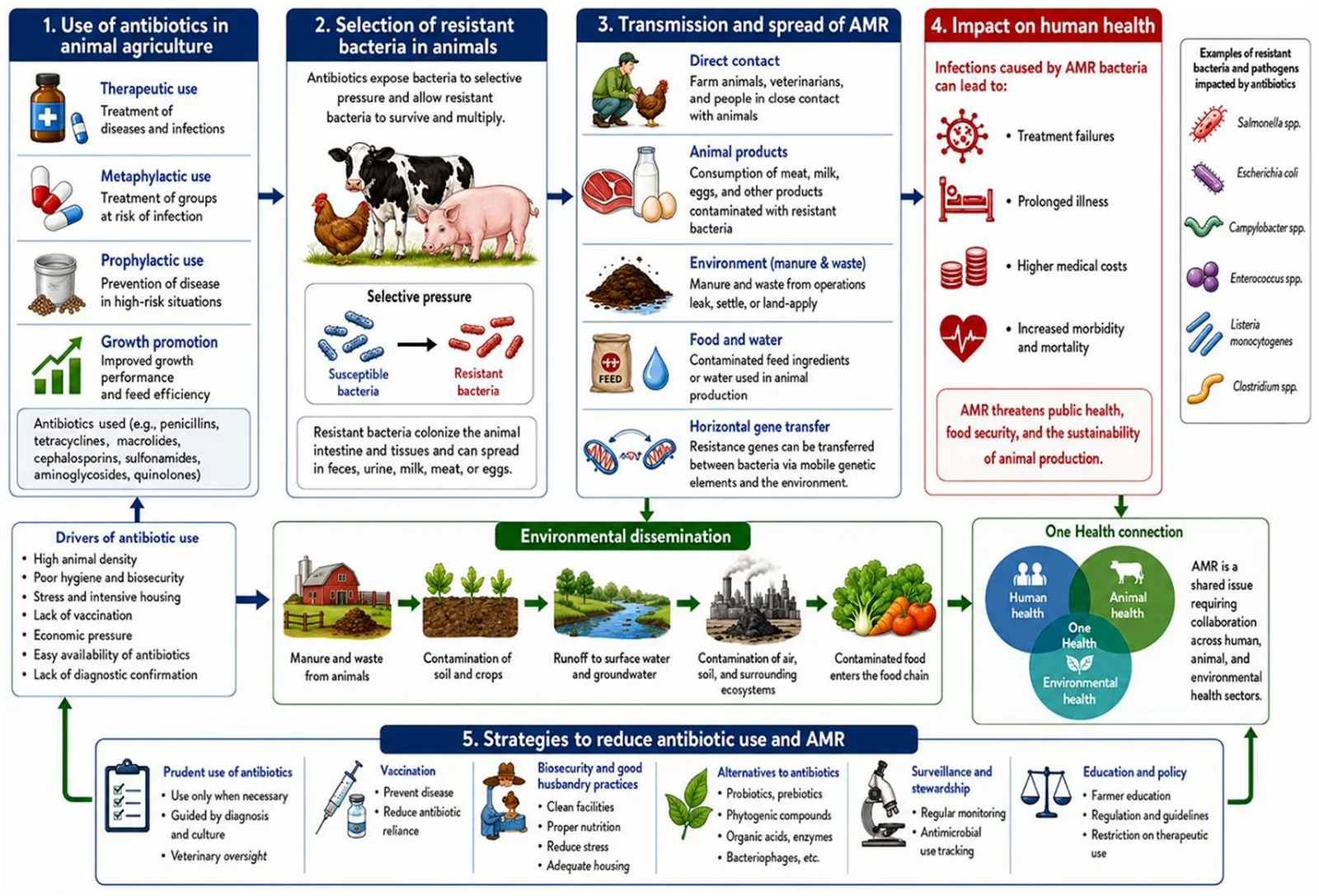
Schematic overview of antibiotic use in animal agriculture and its role in the development and spread of AMR. Illustration created by the authors.

AMR genes and drug-resistant bacteria transfer from food-producing animals to humans through direct contact, consumption of improperly prepared animal products, and environmental exposure (27, 28) (Figure 1). The Centers for Disease Control and Prevention (CDC) estimates that approximately one in five resistant infections in the US originates from animal-derived pathogens (28). Animal waste containing resistant microbes and drug residues serves as a primary environmental reservoir, contaminating water sources and agricultural soils (29). Resistance circulates bidirectionally: animals acquire resistant microorganisms from their surroundings, harbor them in intestines and tissues, and reintroduce them to the environment and human populations (30, 31).

#### 3.2.1 Consumer exposure is substantial

Swiss retail surveillance detected AMR in 22.9% of 38,362 bacterial isolates and 24.6% of 122,438 food samples, with meat products (cuts, ground meat, meatballs) representing 88.7% of resistant samples and 60.1% of AMR-positive bacteria. Isolates included *Enterococcus* spp., *Campylobacter* spp., *Salmonella* spp., *Vibrio* spp., *Listeria* spp., and *E. coli* (32). Livestock products are significant vehicles for extraintestinal MDR pathogens, notably *E. coli* and *Salmonella* spp. (33). Among 152 *Salmonella* isolates from retail animal foods in China, 92.8% exhibited MDR (34).

#### 3.2.2 Milkborne resistance presents additional risks

Balemi et al. (35) identified coagulase-negative staphylococci (39.1%), *S. aureus* (17.2%), *Staphylococcus hyicus* (14.1%), *Staphylococcus intermedius* (9.4%), and *E. coli* (9.4%) in milk from cows, camels, and goats. *E. coli* strains showed MDR to spectinomycin, vancomycin, doxycycline, ceftriaxone, and penicillin; *S. aureus* strains were resistant to penicillin G, spectinomycin, and clindamycin. Swiss surveillance further detected resistant *Enterobacteriaceae* (*Citrobacter* spp., *Cronobacter* spp., *Enterobacter* spp., *Klebsiella* spp., *Shigella* spp., *E. coli, Salmonella* spp.) across milk, cheese, meat, plant, and seafood products, with raw beef and poultry as predominant sources (33). Schematic overview of antibiotic use in animal agriculture and its role in the development and spread of AMR.

## 4 Strategies for reducing antibiotic use in animal production

Addressing AMR requires changes in agricultural practices. According to WOAH, improved animal welfare and husbandry can lower the risk of AMR since stressed animals or those poorly managed are more likely to get infected (36). Thus, the approach to managing AMR in food animals consists of the promotion of good husbandry and biosecurity measures, the timely and evidence-based use of vaccines, responsible use of antimicrobials, responsive governance and regulatory frameworks, comprehensive management of the environment and manure, and education and awareness to reduce the overall use of antibiotics, maintain productivity, and prevent the dissemination of ARB at the animal-human interface (37).

### 4.1 Good husbandry, biosecurity, and farm management

Good husbandry and biosecurity are foundational to reducing antibiotic dependence. WOAH recognizes biosecurity as essential to antimicrobial stewardship (37), while the Codex Alimentarius identifies biosecurity, appropriate nutrition, and vaccination as key measures to reduce antimicrobial need, emphasizing a One Health approach (38). Effective management prevents pathogen introduction, establishment, and spread through controlled animal movement, quarantine protocols, sanitation, pest control, carcass disposal, and continuous health monitoring. These practices reduce infection pressure and improve animal welfare. Complementary measures—adequate housing, optimal stocking density, ventilation, clean water, balanced nutrition, and stress reduction—strengthen host immunity and production efficiency (36, 39).

Reducing antibiotic use is essential to mitigate AMR, but without strong biosecurity and disease control, it can increase animal illness (40). In the U.S., antibiotic-free broiler production rose from <10% in 2014 to nearly 50% by 2018, coinciding with higher incidences of enteric and respiratory diseases such as coccidiosis, clostridial enteritis, colibacillosis, and *Staphylococcus*-associated osteoarthritis and osteomyelitis on poultry farms (41). In swine, diseases like influenza and porcine reproductive and respiratory syndrome (PRRS), along with secondary bacterial infections, remain major drivers of antibiotic use (42). Similarly, bovine respiratory disease (BRD) causes major economic losses in beef cattle (43), while bovine mastitis is the costliest disease in dairy farming and a leading cause of antibiotic treatments (44).

#### 4.1.1 Species-specific applications

- **Poultry**: In the poultry sector, integrated management strategies are especially viable because of the nearly complete vertical integration of production in the US. A single company generally oversees various stages of extensive poultry production, such as feed mills, breeding, hatcheries, grow-out farms, and processing facilities, collectively creating a broiler complex. For example, 90%−95% of broiler and breeder poultry producers and 48% of swine farmers are contracted by large integrators like Tyson Foods and Smithfield, respectively (42). Integrators obtain day-old stock from genetics companies that supply strains selected for desirable traits, with strict standards to keep breeding stock free from infectious diseases (43). Proper handling and storage of hatching eggs are crucial to avoid bacterial contamination. Hatcheries implement sanitation protocols and vaccination for broiler chicks while managing pests to lower disease risks. After hatching, chicks are sent to grow-out farms until they reach processing weight (43). Throughout production, hatchery sanitation, disease-free breeder flocks, proper egg handling, routine chick vaccination, pest control, high-quality nutrition, and optimal environmental conditions (air quality, litter management, and temperature control) collectively reduce disease pressure and the need for antibiotic interventions (37).
- **Swine**: Swine production has advanced disease prevention through improved nutrition for weaned pigs, hygienic housing, and thermal regulation. Air filtration systems in high-density production regions such as Minnesota and Iowa have reduced transmission of porcine reproductive and respiratory syndrome (PRRS). Additional measures include sourcing pigs from high-health breeding herds and vaccination against *Mycoplasma hyopneumoniae*, which has substantially decreased antibiotic use for respiratory diseases since its introduction (45, 46). Denmark further demonstrated that banning antibiotic growth promoters (AGPs) and strengthening herd health programs reduced antimicrobial use by 49% between 1994 and 2017 without compromising productivity (47).
- **Dairy and beef cattle**: In dairy and beef cattle, mastitis prevention relies on proper milking hygiene, teat disinfection, isolation of diseased animals, colostrum management, vaccination against bovine respiratory disease and clostridial infections, and sanitation of feeding and watering equipment. Australia's Countdown program has trained dairy farmers in mastitis prevention and supports selective dry cow therapy combined with internal teat sealants, reducing antibiotic use while maintaining udder health and productivity (48).
- **Small ruminants**: Small ruminants (sheep and goats) are essential for food security in many regions but remain underrepresented in disease-prevention research. Studies from Kosovo identified important gaps in transport hygiene, disease management, and reproduction management, while communal grazing, seasonal transhumance, informal animal trade, and wildlife contact create unique disease transmission pathways that require tailored management approaches (49). Similar findings have been reported in Portugal and Türkiye (50, 51).
- **Aquaculture**: In aquaculture, disease prevention relies on maintaining optimal water quality, appropriate stocking densities, equipment disinfection, health certification of broodstock, and pond or sea-site fallowing between production cycles. Norwegian salmon farming has combined these measures with sea lice monitoring and comprehensive vaccination, reducing antibiotic use by more than 99% since the 1980s. In contrast, Chile continues to use substantially higher quantities of antibiotics, illustrating how differences in disease prevention strategies and regulatory practices influence antimicrobial use (52, 53).

Biosecurity and farm management are most effective when integrated with surveillance systems, evidence-based vaccination programs, prudent antimicrobial use policies, and environmental management of manure and wastewater. Surveillance data inform targeted biosecurity interventions; conversely, robust biosecurity reduces infectious disease burden and antimicrobial demand. While specific biosecurity practices must be adapted to species, production system, and regional context, the underlying principles are universally applicable. From Norwegian salmon farms to Ethiopian smallholder dairies, from vertically integrated U.S. broiler operations to Greek sheep and goat pastoral systems, effective biosecurity and farm management provide the foundation for sustainable animal health, reduced antimicrobial dependence, and AMR mitigation across the human–animal–environment interface.

### 4.2 Vaccination programs

Vaccination plays a crucial role in preventing AMR by reducing infections from both susceptible and resistant bacteria, thereby lowering antibiotic use across food-producing animals. In animals, vaccines have lowered antimicrobial consumption in species including fish, pigs, and poultry (54). By preventing infections, vaccines reduce disease incidence caused by both susceptible and resistant strains, thereby lowering antibiotic use and the selective pressure that drives resistance emergence (55). Thus, using vaccines to reduce antibiotic demand is vital for sustainable animal production. Vaccination is essential for controlling various poultry diseases and is increasingly used to combat coccidiosis as antibiotic-free production increases. There is also a growing trend toward using alternative feed additives such as probiotics and organic acids alongside raised-without-antibiotics (RWA) initiatives (47).

In poultry production, vaccines are essential for controlling various diseases and reducing reliance on antibiotics. Vaccines against coccidiosis caused by *Eimeria* spp. and secondary bacterial infections such as *Clostridium* spp. and *E. coli* exist but face challenges in efficacy and mass delivery. Improving vaccine technologies and administration methods, along with developing more effective vaccines against viral diseases such as infectious bronchitis and infectious bursal disease and their associated secondary bacterial infections, can significantly reduce disease incidence and antibiotic dependence (42). Important antibacterial vaccines—such as those against *Salmonella*—are also key components of integrated disease prevention strategies.

In swine production, vaccines targeting key bacterial pathogens such as *Streptococcus* spp., *Haemophilus* spp., *Pasteurella* spp., *Mycoplasma hyopneumoniae, Actinobacillus* spp., *E. coli, Lawsonia* spp., and *Brachyspira* spp., as well as bacterial infections secondary to viral diseases like PRRS, influenza, and rotavirus, play a crucial role in reducing antibiotic use. However, the development of effective antiviral vaccines is hindered by the high genetic variability of these viruses (56).

In dairy and beef cattle, vaccines against mastitis-causing bacteria (*Staphylococcus* spp., *Streptococcus* spp., *E. coli*) and parasites causing anaplasmosis could substantially lower antibiotic use, since mastitis remains a major source of economic loss and antibiotic treatments in the dairy sector (57).

Vaccine development faces several challenges, including high research and development costs, a lack of reliable models to predict economic returns, and expectations from stakeholders that vaccines provide complete protection. However, stricter antibiotic regulations and growing animal welfare concerns may increase acceptance of partially effective vaccines and encourage market competition to reduce costs (42).

In addition to vaccines, biologic agents and natural products are being investigated as tools to enhance immune function and lower disease incidence. Passive immunization strategies, particularly the use of affordable chicken egg yolk–derived antibodies, have shown potential against enteric diseases such as bovine rotavirus and coronavirus (58). Innate immune modulators, including Imrestor™ (pegbovigrastim) and Zelnate^®^ (plasma-derived myeloid lineage immunostimulant), have been reported to reduce the incidence of mastitis and bovine respiratory disease, respectively, thereby decreasing the need for antimicrobial treatment (59, 60). Natural compounds like essential oils (e.g., carvacrol and eugenol) and medium-chain fatty acids are also under study, though limitations in consistency, dosing, and targeted delivery remain (61). Overall, vaccination remains one of the most effective preventive strategies for reducing disease incidence and antibiotic use, particularly when integrated with biosecurity, surveillance, and antimicrobial stewardship programs.

### 4.3 Prudent antimicrobial use and stewardship

Antibiotic stewardship programs (ASPs) are crucial for addressing AR and ensuring proper use of antimicrobial drugs. CDC defines antibiotic stewardship as “coordinated interventions... designed to improve and measure the appropriate use of antimicrobial agents by promoting the selection of the optimal antimicrobial drug regimen, dose, duration of therapy, and route of administration” (62). These coordinated initiatives aim to promote appropriate antimicrobial use, including antibiotics, with the key objectives of enhancing patient care, minimizing microbial resistance, and controlling the transmission of MDR pathogens (63). These stewardship concepts are equally vital in animal agriculture as they are in human healthcare. There is widespread consensus that antibiotic administration in livestock for therapeutic, preventive, and growth-enhancing purposes contributes to the worldwide increase in AR (10).

On-farm antibiotic stewardship entails the careful and appropriate application of antimicrobial drugs to minimize the emergence and transmission of AR in food animals (64). International case studies demonstrate the effectiveness of such initiatives. For instance, Denmark's prohibition of avoparcin, an antibiotic with similarities to vancomycin, led to substantial reductions in vancomycin-resistant enterococci among both farm animals and people within a 2-year period (65). Similarly, Australia's ban on quinolone use in livestock has produced remarkably low resistance levels in pathogens like *E. coli* and *campylobacter* spp. relative to other nations (37). WHO–commissioned systematic review examining 81 studies found that measures to decrease antibiotic consumption in animals reduced antibiotic-resistant bacteria prevalence by roughly 15% and MDR bacteria by 24%−32% (39). Similarly, human populations demonstrated a 24% reduction in the prevalence of resistant bacteria when antibiotic use was curtailed. Critically, these improvements occurred without negative effects on agricultural productivity or economic returns (65). Furthermore, antibiotic alternatives—including organic acids, probiotics, and vaccines—can decrease antibiotic dependency on farms and strengthen stewardship initiatives (66, 67).

Antibiotic stewardship advancement in the US has lagged behind European efforts. While veterinary authorization for medically important antibiotics in food animals has been mandatory since the 1980s, comprehensive stewardship data collection remains insufficient (68). The FDA's Guidance 209 (2012) and subsequent Guidance 213 (2017) encouraged voluntary responsible use and mandated veterinary oversight for antibiotics delivered through feed or water while prohibiting their use for growth enhancement (69). Nevertheless, the USDA has resisted eliminating medically important antibiotics for disease prevention purposes, permitting continued producer use under preventive classifications (70). Multiple obstacles remain, including insufficient detailed information on clinical justifications, dosing regimens, treatment duration, and farm-specific usage trends, along with weak policy enforcement. Significantly, while antibiotic consumption dropped 30% following Guidance 213 implementation, it rose 8% in the subsequent year, revealing policy shortcomings without more rigorous requirements (71). The US livestock sector utilizes medically important antibiotics at rates 61% higher than 31 European nations collectively. Addressing these stewardship deficiencies requires enhanced surveillance systems, transparent data disclosure, greater veterinary involvement, and improved coordination with public health objectives (37).

Consequently, responsible antimicrobial use and stewardship involve carefully controlled antibiotic administration, rigorous veterinary supervision, and the incorporation of alternative health interventions to reduce antibiotic dependence. These approaches have demonstrated success in lowering resistance rates in both animals and humans while preserving agricultural productivity, emphasizing the essential function of stewardship in maintaining antibiotic efficacy and safeguarding public health.

### 4.4 Regulatory and policy interventions

Regulatory and policy interventions are essential for reducing antibiotic use in animal production and combating AMR. WHO recommends restricting the routine use of medically important antimicrobials in food-producing animals and promotes a One Health approach to AMR control. Similarly, WOAH provides guidelines for the responsible and prudent use of antimicrobials in veterinary medicine. At the national level, many countries have implemented regulations requiring veterinary oversight, antimicrobial surveillance, and prescription-based antibiotic use. In addition, several regions, including the European Union, have banned the use of antibiotics as growth promoters and restricted prophylactic antimicrobial use in livestock. Together, these measures aim to preserve antimicrobial effectiveness while safeguarding animal and public health (72).

### 4.5 Environmental and manure management

Environmental management strategies focus on reducing the release and persistence of antibiotic residues, ARB, and ARGs through improved manure treatment, wastewater management, and waste disposal practices.

#### 4.5.1 Manure management and wastewater management

Anaerobic digestion is an effective and economically viable manure treatment that reduces pathogens, degrades residual antibiotics, and lowers ARGs in manure by up to 90% (61). Thermophilic anaerobic digestion is more efficient than mesophilic systems due to higher operating temperatures, which enhance the reduction of antibiotic-resistant bacteria (73). Composting also decreases active antimicrobial compounds and resistant bacteria primarily through temperature-driven adsorption and degradation. Its effectiveness depends on manure type and composting duration, with reported antibiotic half-lives of up to 16 days, though longer persistence may occur (74). For example, Subirats et al. (75) demonstrated that composting significantly reduced enteric bacteria in broiler chicken litter, including populations carrying AR.

#### 4.5.2 Water quality management

Maintaining water quality is critical for limiting the spread of antimicrobial residues, ARB, and ARGs in aquatic environments. Effective treatment of agricultural wastewater, prevention of manure runoff into surface and groundwater, and regular monitoring of water sources used for livestock production can significantly reduce environmental contamination and AMR dissemination. Improved water management practices also help protect human, animal, and ecosystem health (76).

#### 4.5.3 Reduction of environmental dissemination of ARGs

Effective waste management across households, public spaces, livestock farms, and pharmaceutical industries is essential to limit environmental dissemination of antimicrobials, resistant bacteria, and ARGs. Improper disposal of antimicrobials and chemical residues can create sub-inhibitory concentrations that promote resistance selection (77).

Proper wastewater treatment is essential to limit the environmental spread of AMR, as wastewater represents a major reservoir for both antimicrobial compounds and clinically important ARB (78). Evidence also indicates that reintroducing indigenous microorganisms into agricultural soils can reduce antimicrobial-resistant microbes and ARGs by increasing soil microbial diversity. Klümper et al. (79) reported that native soil microbiota was associated with decreased selection and proliferation of ARGs, likely through mechanisms such as resource competition, antibiotic degradation, production of natural antimicrobials, and reduced horizontal gene transfer.

Preventing AMR in wildlife primarily requires minimizing the release of antimicrobial-resistant microbes and ARGs from human and livestock sources into environments accessible to wildlife. Key control measures include proper disposal of animal carcasses and increasing separation between wildlife, livestock, and humans to reduce opportunities for AMR and ARGs transmission across interfaces (80).

### 4.6 Education and awareness

Educational interventions targeting prescribers, producers, and consumers constitute foundational stewardship components with demonstrated efficacy.

#### 4.6.1 Veterinary education

Curricular integration of antimicrobial pharmacology, resistance mechanisms, and prescribing principles improves prudent use. Continuing professional development requirements maintain competency in diagnostic-based treatment decisions. Veterinary prescribing audits with feedback reduce empirical antibiotic use; analogous programs in livestock medicine show comparable potential (62, 63).

#### 4.6.2 Producer training

Denmark's “Yellow Card” scheme, established in 2010, combines antibiotic use benchmarking with mandatory advisory service consultations and differentiated taxes on critically important antimicrobials. Farms exceeding thresholds receive targeted supervision, and almost all reduce consumption below limits within 9 months. High-consumption weaner herds showed negative growth (reduction) in 82% of cases following implementation (81). Australia's *Countdown Downunder* program, established in 1998, trained over 420 advisers and 1,900 farmers by 2007, with 91% of participants achieving mastitis action plan goals and documented reductions in bulk milk cell counts (82). Training in biosecurity implementation, early disease recognition, and alternative use enables producer autonomy in antibiotic reduction.

#### 4.6.3 Consumer awareness

Public understanding of AMR risks from food animal production remains limited. Campaigns emphasizing proper cooking, food handling, and the environmental footprint of antibiotic use create market pressure for responsible production. Retail certification programs (e.g., antibiotic-free labels) drive industry change but require robust verification to prevent consumer deception. The revised Codex Alimentarius Code of Practice explicitly expands training and communication provisions for all food chain participants (38).

#### 4.6.4 Educational gaps

Smallholder systems in low- and middle-income countries often lack extension services and diagnostic infrastructure. Educational interventions must be culturally adapted, economically feasible, and integrated with practical tool provision (rapid diagnostic tests, decision-support algorithms) rather than being information-only approaches (2, 8).

### 4.7 Integrated implementation

Sustainable mitigation of AMR necessitates the use of multiple interventions. Ensuring good husbandry and biosecurity, vaccinating animals and promoting prudent use of antimicrobials, improving the environment and educating farmers are all effective measures that can help reduce the number of animals that need to be treated. Surveillance is an important part of all of these activities, enabling monitoring and evaluation. The various interventions fit into the overall approach to combating AMR under the one health concept.

## 5 Alternatives to antibiotics in animal production

In response to rising AMR, considerable attention has been given to reducing antibiotic use in livestock production. It is essential to distinguish between performance-promoting alternatives (replacing growth promoters by enhancing gut health and nutrient utilization) and therapeutic alternatives (substituting antibiotics used to treat, control, or prevent clinical infections).

**Figure 2:**
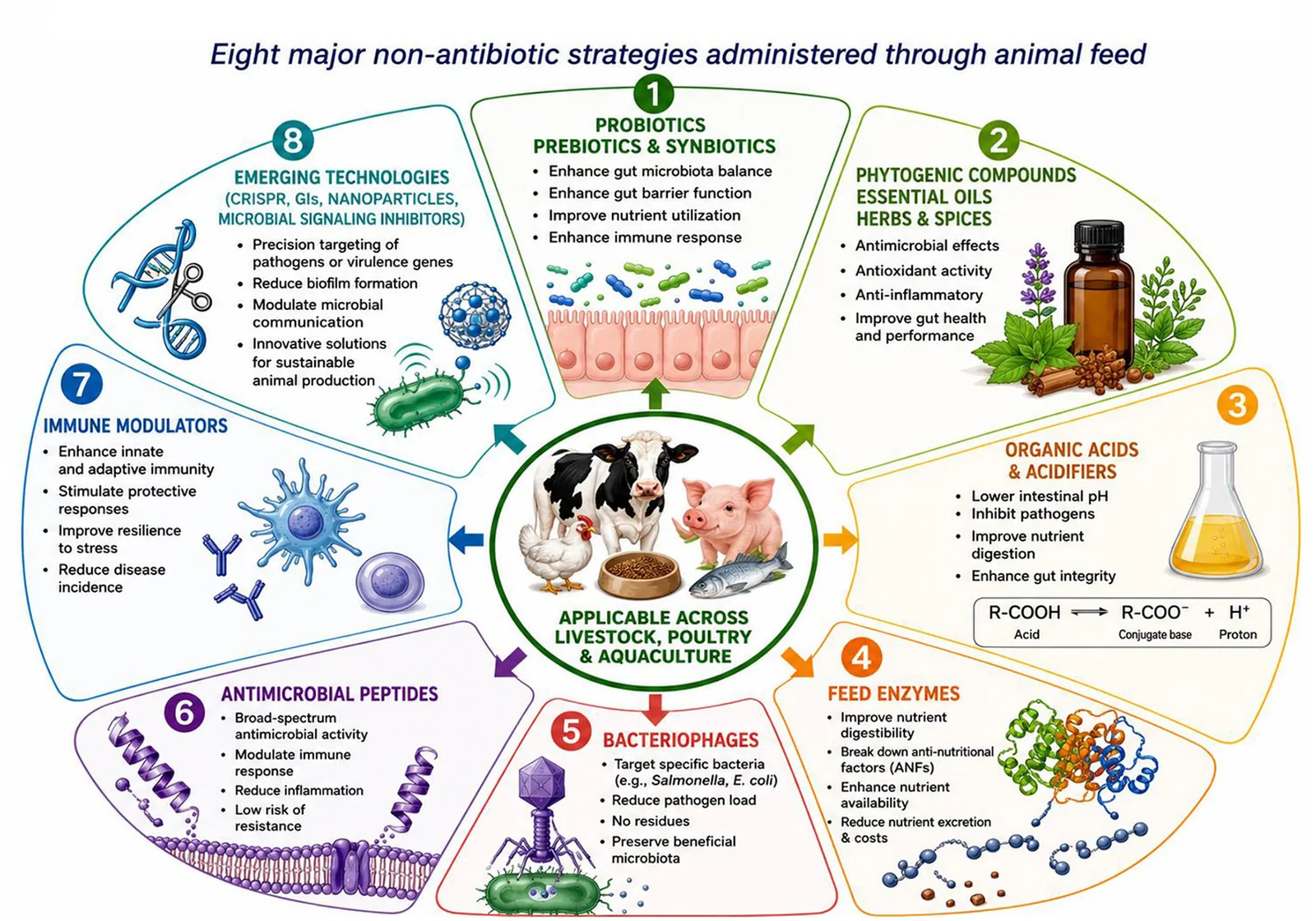
Alternatives to antibiotics in animal agriculture. Illustration created by the authors.

Figure 2 categorizes the major non-antibiotic strategies for reducing antimicrobial use across production systems. No single alternative fully replaces antibiotics; integrated multi-strategy approaches combined with improved husbandry, biosecurity, and vaccination are essential for sustainable reduction.

### 5.1 Probiotics

Probiotics are live microorganisms that confer health benefits when administered in adequate amounts and have emerged as effective alternatives to AGPs in livestock production (83, 84). Their use in poultry, swine, and ruminants has consistently been associated with improved growth performance, feed efficiency, intestinal health, and disease resistance (84–86). Common probiotic microorganisms include species of *Bacillus* spp*., Lactobacillus* spp*., Bifidobacterium* spp*., Enterococcus* spp*., Streptococcus* spp., and *Lactococcus* spp., together with yeasts such as *Saccharomyces* spp. (85, 87, 88).

The beneficial effects of probiotics are primarily attributed to competitive exclusion of enteric pathogens, production of antimicrobial compounds such as bacteriocins, maintenance of intestinal epithelial integrity, and modulation of host immune responses (83, 88–90). In addition, probiotics enhance intestinal barrier function by promoting mucin secretion, maintaining tight junction integrity, supporting epithelial regeneration, and stimulating mucosal immunity, including increased IgA production (89, 90).

Despite these advantages, probiotic efficacy varies according to microbial strain, dosage, diet, animal age, management conditions, and delivery method (87, 91, 92). Commercial application is further influenced by challenges related to product stability during feed processing and storage, survival through the gastrointestinal tract, quality standardization, and the potential transfer of AMR genes from probiotic strains, highlighting the need for careful strain selection and safety evaluation (92, 93).

### 5.2 Prebiotics

Prebiotics are non-digestible dietary carbohydrates, including fructooligosaccharides (FOS), mannan oligosaccharides (MOS), inulin, pectin, and other complex polysaccharides, that are selectively utilized by beneficial intestinal microorganisms to improve gut function and animal performance (94–96). Unlike probiotics, prebiotics act as substrates that support the establishment and metabolic activity of beneficial microbiota rather than introducing live microorganisms.

Through selective microbial fermentation, prebiotics promote a healthier intestinal ecosystem, improve nutrient utilization, and contribute to intestinal integrity (95, 96). Emerging prebiotic sources, particularly seaweed-derived polysaccharides, have shown promising effects on swine health and productivity while reducing dependence on antibiotics (96). In ruminants, selected prebiotics stimulate butyrate-producing microbial populations, thereby supporting rumen epithelial development and function (97). In poultry, dietary prebiotics have also been associated with improved tolerance to heat stress and enhanced meat quality (98).

### 5.3 Synbiotics

Synbiotics are combinations of probiotics and prebiotics designed to improve the survival, colonization, and metabolic activity of beneficial microorganisms in the gastrointestinal tract (99). By supplying both live beneficial microbes and their selective substrates, synbiotics produce complementary effects that are generally greater than those achieved with either component alone.

In livestock production, synbiotic supplementation has been associated with improved nutrient utilization, growth performance, and intestinal health across poultry, swine, and ruminants (87, 100). In ruminants, synbiotics have enhanced growth performance and carcass characteristics (101), whereas in monogastric species they improve gut microbial balance, reduce post-weaning diarrhea and other enteric disorders, and enhance antioxidant status and overall health (102).

An important advantage of synbiotics is their synergistic support of both the intestinal microbiota and host immunity. In poultry, in ovo administration has improved leukocyte development and immune competence, while experimental studies have demonstrated protective effects against MDR pathogens, including *Acinetobacter baumannii* (103). These findings indicate that synbiotics represent a promising strategy for maintaining animal health in antibiotic-reduction programs.

### 5.4 Phytogenic feed additives

Phytogenic feed additives comprise plant-derived products including herbs, spices, essential oils, and plant extracts that are increasingly used as natural alternatives to AGPs in animal production (104). Their biological activities arise from diverse bioactive compounds, including phenolics, terpenoids, alkaloids, flavonoids, and tannins, which collectively contribute to improved animal health and productivity (105). The following sections summarize the characteristics of herbs and spices and essential oils separately.

Various herbal compounds are considered potential alternatives to AGPs for use in food-producing animals (106–108). A variety of phytochemicals, including garlic (*Allium sativum*), turmeric (*Curcuma longa*), cinnamon, *Moringa oleifera*, tannins, saponins, and essential oils, exhibit antimicrobial, antioxidant, anti-inflammatory, and immunomodulatory properties. They can be used to control pathogens, reduce oxidative stress and inflammation, regulate the animal immune response, and improve intestinal health and disease resistance, decreasing the need for antibiotics. Representative studies in poultry, pigs, and ruminants have reported beneficial effects on productivity, intestinal bacteria, and animal health; however, their effectiveness seems to be related to the specific plant, its phytochemical compounds, dose, and production system (109–115).

Despite their potential, limitations of herbs and spices include determining optimal dosage, lipophilicity restricting delivery to gut pathogens, strong odors, inconsistent outcomes due to variability in composition, toxicity at high doses, and high production costs (116, 117).

#### 5.4.1 Essential oils

Essential oils are concentrated volatile extracts obtained from aromatic plants and are rich in phenolic compounds such as thymol, carvacrol, eugenol, and cinnamaldehyde (106, 118, 119).

The antimicrobial efficacy of essential oils stems primarily from their lipophilic nature, which enables attachment to bacterial cell surfaces, penetration and disruption of cell membrane integrity, impairment of cellular metabolism, and ultimately cell death (120). Essential oils exhibit broad-spectrum activity against both Gram-positive and Gram-negative pathogens including *Salmonella* spp*., E. coli, Listeria monocytogenes, Bacillus* spp., *Pseudomonas* spp., and *Clostridium* spp. (121). Essential oils rich in thymol, carvacrol, eugenol and cinnamaldehyde have demonstrated antimicrobial activity against *Listeria monocytogenes, E. coli, Bacillus thermosphacta*, and *Pseudomonas fluorescens* (122). In addition to their direct antimicrobial activity, essential oils may improve intestinal health and reduce pathogen burden, thereby contributing to lower antibiotic use in livestock production (123).

However, practical challenges include volatility leading to rapid degradation, strong odors potentially affecting feed intake, rapid absorption in the proximal gastrointestinal tract limiting distal gut activity, and compositional variability based on plant source, harvest conditions, and extraction methodology (106, 115). Encapsulation technologies and microemulsion formulations are being developed to improve stability, targeted delivery, and sustained release throughout the digestive tract (106, 124).

Table 1 summarizes recent peer-reviewed studies comparing the efficacy of phytogenic feed additives across livestock and aquaculture species, including mixed plant extracts for poultry and ruminants, cinnamon in broilers, essential oil blends in weanling pigs, saponin-rich plants in ruminants, and bioactive compounds in fish diets.

**Table 1:** Broad-spectrum benefits of phytogenic feed additives across livestock.

Phytogenic feed additive	Active compound	Species	Key findings	References
Mixed plant extracts	Phytogenic additives	Poultry & ruminants	Improved antioxidant status, educed toxin effects, and enhanced gut protection.	(197)
Cinnamon supplementation	Cinnamon	Poultry	Enhanced growth, stronger immune response, reduced pathogenic bacteria.	(198)
Essential oil blends	Essential oils	Weanling pigs	Improved intestinal morphology, increased beneficial microbes, better performance.	(199)
Saponin- rich plants	Plant saponins	Ruminants	Reduced methane production, improved microbial fermentation profile.	(200)
Bioactive compounds	Phytogenic bioactive compounds	Fish	Enhanced mucosal immunity, increased disease resistance, improved gut health.	(201)
Black pepper or radish seed oils	Essential oil blend	Broiler chickens	Modulated performance and expression of digestive enzymes, lipogenesis, immunity, and autophagy-related genes	(104)
Selected African medicinal plants (Euclea, Ficus, Aloe, Lippia, Artemisia)	Medicinal plant extracts	Livestock (general)	Promising solutions against multi-drug resistant bacteria	(105)
Thymol	Essential oil (phenolic compound)	Swine	Improved barrier function, attenuated inflammatory responses during LPS-induced inflammation	(106)
*Scrophularia striata* and *Ferulago angulate*	Herbal extracts	Broiler chickens	Alternative to virginiamycin; improved growth performance, intestinal microbial population, immune response, blood components	(107)
Tannins	Polyphenol phytogenics	Livestock (general)	Alternative to antibiotics; suppressed pathogenic microorganisms, reduced oxidative stress and inflammation, modulated immune responses	(108)
Garlic (*Allium sativum*)	Herb/spice	Poultry, Swine, Beef, Dairy	Broad-spectrum antibacterial activity against Gram-positive and Gram-negative pathogens; synergistic effects with conventional antibiotics	(109)
Turmeric (*Curcuma longa*)	Herb/spice	Swine (weanling piglets)	Improved growth performance, enhanced gut health, promoted short-chain fatty acid production	(110)
Cinnamon Powder(*Cinnamomum zeylanicum*)	Herb/spice	Broiler chickens	Improved body weight gain, feed efficiency, hematological parameters, and beneficial gut microbiota shifts.	(111)
In ovo polyphenol-rich herbal compounds (grape pomace, resveratrol, chicoric acid)	Polyphenol	Poultry (in ovo)	Improved hatchability, chick quality, post-hatch performance; reduced oxidative stress and infection susceptibility.	(112, 113)
*Moringa oleifera* leaf extract	Herb/Spice	Poultry	|Anti-coccidial, antibacterial, immune-modulating, and growth-enhancing properties.	(114, 115)
Cinnamaldehyde feed additive	Essential oil (aldehyde)	Dairy cattle (lactating)	Improved feed use-efficiency.	(123)

### 5.5 Organic acids

Organic acids are gaining recognition as effective alternatives to AGPs in poultry, particularly with the shift toward antibiotic-free production systems (40). These acids—including formic, acetic, propionic, butyric, and lactic acids—are short-chain fatty acids that can be directly added to feed or produced by microbial fermentation in the gastrointestinal tract. Historically employed for feed sanitization and *Salmonella* spp. control, organic acids now serve broader roles in reducing enteric pathogen loads, improving growth performance, and managing dysbacteriosis—a condition of bacterial overgrowth that has become increasingly prevalent following AGPs withdrawal (125).

Organic acids improve gut health through dual mechanisms. Indirectly, they lower pH in the proximal gastrointestinal tract (crop and gizzard), creating an environment hostile to pH-sensitive pathogens including *Salmonella* spp. and other Gram-negative bacteria (40). Directly, undissociated organic acid molecules penetrate bacterial cell walls and release protons intracellularly, disrupting bacterial metabolism, inhibiting replication, and interfering with protein synthesis—independent of overall gut pH reduction. Organic acids also enhance proteolytic enzyme activity, nutrient digestibility, and support beneficial bacteria growth, stabilizing gut microbial populations and improving gut morphology (126).

However, their effectiveness depends on factors such as timing, acid type and concentration, the health status of the birds, and diminishing acid levels along the digestive tract. Some organic acids may exert adverse effects on beneficial *Lactobacillus* spp. populations at high concentrations, requiring careful optimization of supplementation strategies (125).

### 5.6 Acidifiers

Unlike individual organic acids, acidifiers are commercial feed additives formulated from one or more organic acids, their salts, buffered forms, or encapsulated derivatives to improve stability, handling, and targeted gastrointestinal delivery (127). Their formulation allows sustained release throughout the digestive tract and improves practical application under commercial production conditions.

In poultry and swine production systems, acidifier supplementation has been associated with reduced therapeutic antibiotic use, improved average daily gains and feed conversion ratio (FCR), enhance gut health and immunity, inhibit pathogens, and improve meat quality (127). Encapsulation technologies, including microencapsulated organic acid mixtures, improve stability and enable targeted release throughout the gastrointestinal tract, resulting in enhanced nutrient utilization and favorable carcass characteristics such as reduced drip loss, increased lean meat percentage, and improved backfat thickness in growing-finishing pigs (124).

Acidifier efficacy, however, varies considerably depending on multiple interacting factors: specific acid type and chemical form, pKa and molecular weight, target pathogen species, animal species and age, diet composition and buffering capacity, and underlying animal health status (87, 128, 129). Certain acids or blends may impair rather than improve performance: sulfuric acid can induce electrolyte imbalance and reduce feed efficiency in weaned pigs; phosphoric acid may lower gut pH without corresponding improvements in nutrient utilization; and mixed inorganic-organic formulations have shown inferior growth performance compared to organic acids alone (130, 131). Overuse of acidifiers can negatively affect feed palatability, cause tissue irritation or damage, and create handling difficulties because of their corrosive and volatile nature; therefore, encapsulation is often required to achieve controlled release (87). High doses of specific acids, including benzoic acid, have been associated with functional impairment and structural damage to organs such as the liver, spleen, and lungs, alongside adverse intestinal morphological changes (132). To mitigate these challenges, commercial formulations increasingly employ encapsulation using fatty acids or lipid matrices to achieve controlled, section-specific release within the gastrointestinal tract. Despite promising efficacy, the limited availability of rigorous economic evaluations comparing acidifiers to conventional antimicrobials constrains evidence-based adoption decisions in commercial settings (133).

### 5.7 Feed enzymes

Exogenous feed enzymes are effective alternatives to antibiotic growth promoters, particularly in poultry and swine, because they improve nutrient utilization by degrading anti-nutritional factors such as non-starch polysaccharides, phytate, and poorly digestible proteins (134). Enhanced nutrient availability reduces substrates available for pathogenic bacteria, supports intestinal health, improves feed efficiency, and lowers nutrient excretion without leaving residues in animal-derived products (135).

The most widely applied feed enzymes include phytases, carbohydrases (xylanase, β-glucanase, cellulase), proteases, and glycanase blends, with phytase being the most commonly incorporated single enzyme in monogastric diets (134). Recombinant enzyme technologies have enhanced activity and stability under feed processing conditions. Phytase improves phosphorus and mineral bioavailability while positively influencing gut microbiota composition. Xylanase supplementation reduces *Clostridium perfringens*-associated enteric diseases in broiler chickens by degrading viscous arabinoxylans that promote pathogen colonization (136). Encapsulated lysozyme and other targeted enzyme formulations have demonstrated efficacy in reducing pathogen loads and associated intestinal damage (136).

Supplementation with single or multi-enzyme preparations has consistently improved nutrient digestibility and feed efficiency across poultry, swine, and ruminants. In monogastric animals, enzyme blends enhance intestinal morphology and beneficial microbial populations, whereas fibrolytic and amylolytic enzymes improve fiber degradation, rumen fermentation, milk yield, and feed efficiency in dairy cattle (134, 137–139).

However, enzyme efficacy is influenced by multiple variables including basal diet composition and ingredient quality, animal genetics and physiological stage, enzyme source and formulation, processing conditions (pelleting temperature, conditioning time), and gastrointestinal stability (139). The impact of in-feed enzymes on growth performance remains inconsistent across studies, and concerns persist regarding the ability of exogenous enzymes to withstand feed manufacturing temperatures and maintain activity throughout transit through the digestive system.

## 6 Emerging and innovative alternatives

Emerging alternatives to conventional antimicrobials represent an active area of research for addressing AMR in animal production (57, 120). Current technologies under investigation include bacteriophages, antimicrobial peptides, quorum-sensing inhibitors, host-targeted immunomodulators, CRISPR-Cas–based antimicrobials, nanoparticle-based antimicrobial delivery systems, and artificial intelligence (AI) for disease surveillance, rapid diagnostics, antimicrobial discovery, and precision decision-making (57, 120). These technologies aim to improve pathogen control while reducing reliance on conventional antibiotics (42, 130). Although promising, most remain at the experimental or early translational stage and require further validation through controlled field studies, safety and efficacy assessments, regulatory approval, and economic evaluation before they can be recommended for routine antimicrobial stewardship or antimicrobial use management in livestock production (57, 120).

### 6.1 Bacteriophages

Bacteriophages have emerged as a strategic and effective alternative to antibiotics within the livestock and poultry sectors (140). Over the last 20 years, there has been significant progress in research and regulatory frameworks surrounding the use of bacteriophages in regions such as China, the US, and the European Union. Organizations like the China Phage Research Alliance and the European Medicines Agency have established guidelines to ensure safety, efficacy, and quality for both clinical and veterinary applications (141).

In comparison to other alternatives to antibiotics, bacteriophages present distinct advantages. These include greater diversity and selectivity, the ability to specifically target host bacteria without disrupting normal microbiota, and their capacity to co-evolve with bacterial hosts, which helps in preventing resistance development. Furthermore, they do not replicate within eukaryotic cells, reducing potential toxicity concerns for animals (142, 143).

In practical terms, bacteriophages are often administered as cocktails that combine multiple phages to address a wide array of bacterial pathogens while minimizing resistance risks. Research indicates that incorporating bacteriophage cocktails into livestock feed can enhance growth performance, improve intestinal barrier integrity, modify gut microbiota by promoting beneficial bacteria such as *Lactobacillus* spp. and *Bifidobacterium* spp., and decrease harmful bacteria like *Salmonella* spp*., E. coli, Clostridium perfringens*, and *Clostridium difficile*. This ultimately leads to reduced inflammation and lower rates of diarrhea incidence (140).

Building on these strategic advantages, Table 2 compiles the key *in-vivo* evidence for phage-driven replacement of antibiotics in animal industry.

**Table 2:** Bacteriophage as an antibiotic alternative in the animal industry.

Species	Phage cocktail (dose; target pathogens)	Key Findings	Reference
Weaned piglets	400 mg/kg feed; *Salmonella, E. coli, Clostridium perfringens, Staphylococcus aureus*	•Increased Average daily feed intake •Improved the integrity of the intestinal barrier •Inhibiting intestinal inflammation and reducing the rate of diarrhea in piglets	(202)
Weaned piglets	1.5 g/kg feed; broad-spectrum	•Increased ileal *Lactobacillus* spp. •Reduced content of E. coli (5.76 %) and C. difficile (4.21 %) •*Lactobacillus* spp. fermentation lowers intestinal pH, curbing pathogens and stabilizing gut flora	(203)
Growing pigs	Linear dose (0–1 g/kg feed); broad-spectrum	•Linear increase in daily weight gain and apparent digestibility •Increases faecal *Bifidobacterium* and *Lactobacillus* •Decrease C. *perfringens* and *E. coli* in feces	(204)
Broilers (*Salmonella*-challenged)	vB_SenM-2 +vB_Sen-TO17 in drinking water	•Normalized haematology and plasma ALT activity •Curbed *Salmonella* replication and concurrently eased hepatic metabolic load	(205)
Laying hens	10 mg/kg feed; anti-S*almonella*	•25.58% reduction in caecal *Salmonella* counts •14.74 % reduction in faecal *Salmonella* counts	(206)
Broilers	500 mg/kg feed (DM bases)	•Increase ileal *Bifidobacterium, Prevotella* and L. salivarius •Decrease *Lactobacillus aviarius* vs antibiotics	(207)

Across these studies, multi-phage “cocktails” proved consistently effective in replacing in-feed antibiotics by selectively suppressing targeted pathogens while preserving or enhancing beneficial gut microbiota, improving zootechnical performance, and avoiding the metabolic side-effects observed with conventional drugs.

### 6.2 Antimicrobial peptides

Antimicrobial peptides (AMPs) are small bioactive peptides that constitute an important component of innate immunity and are derived from microorganisms, plants, insects, animals, and humans (144). Compared with naturally occurring peptides, synthetic AMPs generally exhibit greater antimicrobial potency, improved stability, lower toxicity, and increased resistance to proteolytic degradation. Representative AMPs include bacteriocins, defensins, protegrins, tachyplesins, and insect defensins (145).

Studies indicate that antimicrobial peptides contribute positively to growth performance, nutrient retention, intestinal histomorphology, and the balance of gastrointestinal microbiota (146, 147) AMPs also modulate innate and adaptive immune responses by regulating inflammatory mediators, thereby improving intestinal health and disease resistance (148, 149). Dietary microcin J25 has demonstrated protective effects against *E. coli* and *Salmonella* spp. infections in broiler chickens while improving intestinal morphology and reducing intestinal inflammation, with similar benefits reported in piglets (150, 151). Similar outcomes were noted with the addition of microcin J5 to piglet diets (152). AMPs exert antimicrobial activity primarily by disrupting bacterial cell membranes, resulting in membrane permeabilization, leakage of intracellular contents, and eventual cell lysis (87).

Despite their potential, the application of antimicrobial peptides (AMPs) is limited by reduced stability during feed processing and storage, limited gastrointestinal bioavailability, and the potential for bacterial resistance development following prolonged use (148, 150–152). Therefore, further optimization of peptide formulation, delivery systems, and safety evaluation is required before AMPs can be widely adopted as routine alternatives to conventional antibiotics in livestock production (145).

### 6.3 Quorum-sensing inhibitors (QSIs)

Quorum sensing (QS) is a bacterial cell-to-cell communication system that coordinates collective behaviors in response to population density. Pathogens use QS to regulate the expression of virulence factors, biofilm formation, and ARGs, thereby facilitating infection and disease transmission. QSIs disrupt these signaling pathways, reducing bacterial virulence without directly killing bacteria and thus potentially minimizing selective pressure for resistance development (109, 153).

Several limitations currently constrain the practical application of QSIs in animal production. Because quorum-sensing signals such as autoinducer-2 (AI-2) are also involved in communication among beneficial gut bacteria, broad QSI application may disrupt microbial homeostasis (154). Furthermore, QS inhibition does not consistently reduce virulence and, in some pathogens, may enhance biofilm formation or persistence (155–157). The potential for resistance to QSIs has also been reported, highlighting the need for pathogen-specific targeting and comprehensive evaluation of efficacy, safety, and long-term impacts. Consequently, QSIs should currently be regarded as investigational approaches requiring further validation before routine application as alternatives to antibiotics in livestock production (154, 158).

### 6.4 Immune modulators

Immune modulators, such as synthetic oligodeoxynucleotides containing CpG motifs, invoke a passive immune response through the transfer of antibodies. Unlike vaccines, these modulators activate the immune system in a manner that is less reliant on the specific pathogen responsible for an infection, allowing them to be effective against a wide variety of pathogens. Research has indicated that synthetic CpG-containing oligodeoxynucleotides (CpG ODN) or bacterial DNA exhibit protective immunological effects against extracellular bacterial infections in poultry. CpG ODNs enhance the immune system's ability to generate a rapid innate immune response and promote T helper 1 immunity (10).

In poultry, meta-analytic evidence demonstrates that egg-yolk antibodies significantly reduce necrotic enteritis risk (159), while CpG-based immune modulators attenuated *E. coli*-induced clinical symptoms in challenged broiler chicks (160). In swine, egg-yolk antibodies showed efficacy against bacterial and viral enteric pathogens (159), though glycan-based modulators yielded inconsistent results due to limited data (161). Vitamin C and glycans demonstrated promising outcomes against bacterial toxins in young piglets (162), yet egg-yolk antibody application is constrained by gut stability, narrow host spectrum, and high production costs (163). In the US, two immune modulators have achieved regulatory approval for cattle: a cytokine-based product for post-calving mastitis prevention (FDA-approved) (164) and a CpG-based biologic for bovine respiratory disease (USDA-approved) (165).

Efficacy of immunostimulants depends on immune competence, limiting utility in neonates or severely stressed animals. Safety concerns regarding developmental effects in immature immune systems, coupled with poorly characterized mechanisms of action, necessitate cautious application.

### 6.5 CRISPR-Cas system

The CRISPR-Cas (clustered regularly interspersed short palindromic repeats-CRISPR associated protein) adaptive immune system of bacteria employs DNA-encoded, RNA-mediated, or DNA-targeting processes that hinder the attack of bacteria by mobile genetic elements such as phages and plasmids and other foreign genetic material. With the help of these gene-editing technologies, it is possible to target bacterial genomes quantitatively, specifically, and selectively to diminish or completely eradicate AR and open up new therapeutic avenues for MDR illnesses (120).

However, current evidence for CRISPR-Cas-based therapeutic elimination of resistance in animal production remains extremely limited; the technology has been predominantly validated for diagnostic applications rather than *in vivo* antimicrobial intervention (166). CRISPR-Cas diagnostics for AMR gene detection require upstream nucleic acid amplification—such as recombinase polymerase amplification (RPA) or loop-mediated isothermal amplification (LAMP)—to achieve clinically relevant sensitivity, introducing workflow complexity, contamination risks, and dependence on sample preparation free of PCR inhibitors including hemoglobin, immunoglobulins, and mucopolysaccharides (166). Off-target binding remains a concern due to mismatch tolerance in Cas enzymes, particularly problematic given the sequence homology among resistance gene families such as β-lactamase variants (166). Furthermore, the Protospacer Adjacent Motif (PAM) requirement constrains targetable sequences—for instance, SpCas9 requires 5′-NGG-3′ PAM, limiting approximately one-third of all possible target sites—and the collateral cleavage signal used for detection suffers from saturation kinetics that limit quantitative dynamic range (167). While SHERLOCK and DETECTR platforms have achieved FDA Emergency Use Authorization for viral detection, regulatory validation for veterinary AMR applications is still emerging, and phenotypic confirmation remains essential since genotypic resistance does not invariably correlate with expressed phenotypic resistance (166). Consequently, CRISPR-Cas should be regarded as a future, complementary diagnostic approach rather than an established therapeutic strategy for antimicrobial stewardship in animal production.

### 6.6 Nanotechnology-hased interventions

Nanotechnology plays a vital role in developing novel antimicrobial strategies by enhancing interaction with bacteria and boosting bioavailability, absorption, and mucoadhesion. The distinctive physicochemical characteristics of nanoparticles (NPs), such as their high surface-to-volume ratio and capacity to cross biological barriers, render them especially potent against resistant pathogens (168). Metal NPs, particularly silver (AgNPs), gold (AuNPs), and copper (CuNPs), exert bactericidal effects via reactive oxygen species generation, membrane disruption, and interference with bacterial respiration—mechanisms distinct from conventional antibiotics that mitigate cross-resistance risk (169). In livestock systems, AgNPs reduce *Salmonella* spp. and *E. coli* colonization in poultry, while CuNPs demonstrate growth-promoting properties in swine comparable to AGPs (170). In poultry and swine, dietary ZnO-NPs at pharmacological doses have shown growth-promoting and anti-diarrheal effects comparable to AGPs (171). AgNPs have also been investigated for topical wound healing and mastitis treatment in dairy cattle. Nano-encapsulation further enables targeted delivery of phytogenic compounds and antimicrobial peptides to the distal gastrointestinal tract (172). Despite promising *in vitro* and preclinical findings, clinical translation remains limited due to cytotoxicity concerns, tissue accumulation, environmental persistence, and inadequate regulatory frameworks for food-producing animals (173). Although nanotechnology exhibits greater technological readiness than CRISPR-Cas or phage therapy—owing to established applications in medical coatings and wound dressings—its integration into livestock production requires further safety validation and species-specific guidelines before recommendation as a stewardship strategy (168).

### 6.7 Artificial intelligence and machine learning applications

Artificial intelligence (AI) has transformed the management of infectious diseases by improving pathogen detection, recognizing resistant bacteria, and aiding in target identification, dynamic modeling, peptide design, and drug repurposing, thereby bolstering antibiotic stewardship and addressing AMR (174, 175). Machine learning (ML) algorithms analyze large-scale epidemiological, genomic, and phenotypic datasets to identify resistance determinants and forecast resistance trends in livestock populations. Deep learning architectures, including convolutional and recurrent neural networks, enable automated interpretation of antimicrobial susceptibility testing and rapid classification of resistant phenotypes (176).

In veterinary contexts, AI-driven decision support systems assist practitioners in selecting optimal antimicrobial therapies by integrating herd-specific health records, local resistance profiles, and pharmacokinetic parameters (177). Predictive models trained on farm-level data can identify high-risk periods for disease outbreaks, enabling targeted metaphylactic interventions and reducing unnecessary antibiotic use (178). AI algorithms also accelerate the discovery of antimicrobial peptides and phytogenic compounds with activity against MDR livestock pathogens (179, 180). Furthermore, AI-powered image analysis facilitates early detection of mastitis, respiratory disease, and lameness in livestock, supporting timely intervention and reducing empirical antimicrobial use (181–183). Despite these advances, AI implementation remains constrained by limited standardized datasets, interoperability challenges, and the need for validation across diverse production systems (184, 185). Consequently, AI should be viewed as a complementary decision-support tool that augments, rather than replaces, veterinary expertise within antimicrobial stewardship programs (186, 187).

### 6.8 Other alternatives

A variety of alternative products, including heavy metals and clay minerals, have been proposed as substitutes for antibiotic growth promoters, some with concurrent disease prevention properties.

#### 6.8.1 Zinc, copper, and other heavy metals

Zinc, copper, and other heavy metals are essential trace elements that are commonly supplemented at pharmacological doses for growth promotion and occasional enteric disease therapy (188). The European Commission has concluded that copper promotes growth in broiler chickens and swine (189), and meta-analyses have demonstrated growth-enhancing effects of zinc oxide in piglets (190) and copper in beef cattle (191), though the Commission noted copper is not established as a growth promoter in species other than pigs and chickens and can reach toxic levels in calves (192). Inorganic mineral combinations including copper, iron, manganese, and zinc significantly improved weight gain in broilers (193). Copper supplementation has also improved laying hen performance, and zinc oxide reduced post-weaning diarrhea incidence in pigs (190). However, concerns regarding heavy metal residues in meat require careful consideration (194). Additionally, heavy metal use for growth promotion may co-select for AR due to genetic linkage of resistance genes on shared plasmids (195).

#### 6.8.2 Clay minerals and rare earth elements

Other proposed growth-promoting substances include clay minerals (e.g., bentonites, zeolites) and rare earth elements (e.g., scandium, lanthanum) (66). While some demonstrate growth-promoting or disease-preventive potential, efficacy data remain limited and often conflicting, and safety data are frequently inadequate.

## 7 Future directions and research gaps

No single alternative to antibiotics currently satisfies all requirements of non-toxicity, stability, environmental sustainability, and equivalent efficacy to conventional antimicrobials. Reducing antibiotic usage without concomitant improvements in farming practices risks higher infection rates and increased dependence on higher-tier antibiotics (e.g., cephalosporins, carbapenems) or combination therapies, which are more costly and accelerate resistance development (152).

Current vaccines, probiotics, phytogenics, organic acids, and feed enzymes each demonstrate partial efficacy but cannot individually replicate the combined therapeutic and growth-promoting functions of conventional antibiotics in livestock production. Vaccines primarily prevent infectious diseases and thereby reduce the need for therapeutic, metaphylactic, and prophylactic antibiotic use, whereas other alternatives improve gut health, nutrient utilization, immune function, or pathogen control through complementary mechanisms. Challenges—including inconsistent efficacy across studies and production systems, narrow antimicrobial spectra, formulation and delivery limitations, scalability constraints, and incompletely characterized mechanisms of action, continue to limit their widespread adoption. Research priorities should include elucidating their effects on gut microbiota, pathogen colonization, biofilm disruption, host immunity, and optimized delivery systems for phytochemicals, essential oils, and other bioactive compounds.

Several innovative antimicrobial technologies are emerging as promising tools for combating AMR in animal production systems. Nanotechnology-based interventions, metal and metal oxide nanoparticles, bacteriophage therapy, quorum-sensing inhibitors, and CRISPR/Cas-based genome-editing technologies have been discussed in the preceding section and represent promising future approaches for combating AMR in animal production systems. However, these technologies have not yet been sufficiently validated under field conditions, and their safety, long-term efficacy, environmental impacts, economic feasibility, and practical implementation remain largely unproven. Consequently, they should be regarded as investigational approaches rather than current strategies for antimicrobial stewardship or antimicrobial use management strategies until supported by robust experimental and field evidence.

Future research should prioritize integrated antimicrobial stewardship approaches that incorporate improved husbandry, biosecurity, vaccination, environmental management, precision nutrition, microbiome-based interventions, and advanced surveillance systems (196). Long-term field studies evaluating efficacy, economic feasibility, scalability, sustainability, and applicability across diverse livestock production systems are essential to support evidence-based implementation and strengthen global antimicrobial stewardship.

## 8 Conclusion

AMR in the animal industry poses a profound threat to animal health, food safety, public health, and environmental sustainability. This review demonstrates that excessive antimicrobial use in livestock production is a major driver of resistance development and dissemination, necessitating urgent and coordinated antibiotic reduction strategies. Evidence from global regulatory experiences and farm-level interventions confirms that meaningful reductions in AMR are achievable when integrated antimicrobial stewardship incorporates improved husbandry, biosecurity, vaccination programs, environmental management, and the judicious use of validated antibiotic alternatives. No single alternative can fully replace antibiotics; instead, a multifaceted and integrated approach is essential. Preventive strategies that enhance animal immunity, gut health, and overall resilience rather than merely substituting antibiotic functions represent the most sustainable pathway forward. Emerging technologies such as bacteriophages, antimicrobial peptides, and gene-editing tools offer promising future solutions, but their success will depend on continued research, regulatory clarity, economic feasibility, and producer acceptance. Importantly, effective AMR mitigation must be framed within a One Health perspective, recognizing the interconnectedness of humans, animals, and ecosystems. Strengthening surveillance systems, harmonizing international policies, improving data transparency, and fostering collaboration among farmers, veterinarians, researchers, industry stakeholders, and policymakers are critical to achieving long-term success. Ultimately, reducing antimicrobial reliance in animal production is not only feasible but essential to safeguarding antibiotic efficacy, ensuring sustainable livestock systems, and protecting global public health for future generations.
